# Serum lipoprotein(a) concentrations in presumably healthy Polish subjects in relation to age, sex, and cardiometabolic risk factors

**DOI:** 10.11613/BM.2026.010702

**Published:** 2025-12-15

**Authors:** Anna Stefańska, Katarzyna Bergmann, Łukasz Szternel, Joanna Siódmiak, Aleksandra Wolska, Blanka Dwojaczny, Magdalena Krintus, Mauro Panteghini

**Affiliations:** 1 Department of Laboratory Medicine, Nicolaus Copernicus University in Torun, Collegium Medicum in Bydgoszcz, Poland; 2 Department of Human Physiology, Nicolaus Copernicus University in Torun, Collegium Medicum in Bydgoszcz, Poland

**Keywords:** lipoprotein(a), cardiovascular risk, lipoproteins, dyslipidemia, epidemiology

## Abstract

**Introduction:**

Lipoprotein(a) (Lp(a)) is an independent cardiovascular risk factor, primarily determined by genetic factors. This study assessed Lp(a) concentrations in presumably healthy subjects and evaluated its association with age, sex, and cardiometabolic risk factors.

**Materials and methods:**

The study included presumably healthy 1046 adults and 276 children. Laboratory parameters: lipid profile, Lp(a), apolipoprotein B (apoB), glucose, HbA1c, C-reactive protein and creatinine were measured. Contributions of Lp(a)-apoB to apoB (%Lp(a)/apoB) and of Lp(a)-cholesterol to LDL-cholesterol (%Lp(a)-C/LDL-C) were calculated.

**Results:**

Lipoprotein(a) concentrations were significantly higher in adults than in children (P = 0.014) and in women than in girls (P = 0.003), but showed no overall sex differences. In women, Lp(a) was higher after age 50, while in men a slight rise occurred after age 60. Lipid indices %Lp(a)/apoB and %Lp(a)-C/LDL-C declined in men until their 40s and was higher after 50 in both sexes. In a multivariable logistic regression model increased LDL-C concentration was a significant predictor of Lp(a) ≥ 0.30 g/L in women (odds ratio, OR = 1.77; P = 0.021) and children (OR = 2.83; P = 0.009). Boys had twofold higher probability of Lp(a) ≥ 0.30 g/L than girls (OR = 2.17; P = 0.024).

**Conclusions:**

Lipoprotein(a) concentrations increase with age, especially after 50 in women and 60 in men, and are significantly associated with LDL-C. Rising %Lp(a)/apoB and %Lp(a)-C/LDL-C alongside falling apoB and LDL-C suggest greater atherogenicity in older individuals, particularly men. These findings support including Lp(a) in lipid profile for better cardiovascular risk assessment.

## Graphical abstract

**Figure:**
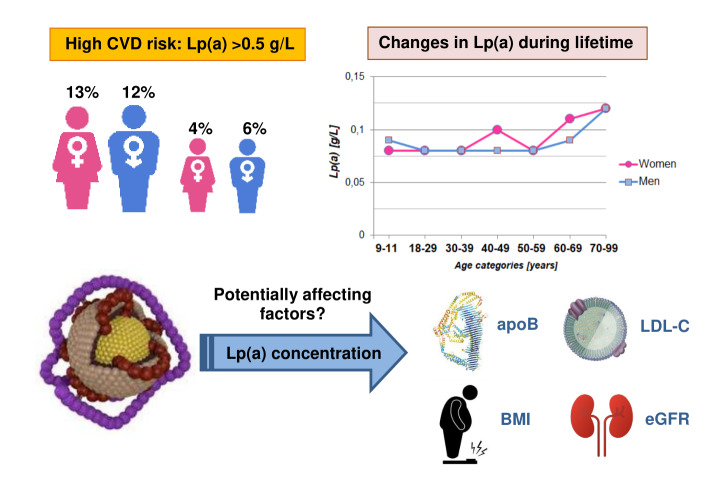

## Highlights

• The first Polish study evaluating lipoprotein(a) (Lp(a)) concentrations in both adults and children

• The first Polish study including Lp(a)-dependent lipid indices: %Lp(a)/apoB and %Lp(a)-C/LDL-C

• We evaluate the association of Lp(a) with age, sex and cardiometabolic risk factors

• We found a significant impact of age and LDL-C on Lp(a) concentration

## Introduction

Lipoprotein(a) (Lp(a)), a low-density lipoprotein (LDL)-like lipoprotein containing apolipoprotein(a), is an independent risk factor for cardiovascular disease (CVD), including coronary artery disease (CAD), myocardial infarction, stroke and peripheral arterial disease (*1*). Guidelines have classified serum Lp(a) concentrations for cardiovascular risk assessment as low (< 0.30 g/L; < 75 nmol/L), moderate (0.30-0.50 g/L; 75-125 nmol/L), high (0.50-1.80 g/L; 125-450 nmol/L), and very high (> 1.80 g/L; > 450 nmol/L), respectively (*1*, *2*). They advocate for a single lifetime Lp(a) measurement as part of comprehensive CVD risk evaluation, emphasizing its role in identifying individuals at underestimated risk, particularly those with high and very high concentrations. Lipoprotein(a) measurement is strongly recommended in subjects with a personal or family history of premature cardiovascular events (*1*, *2*). Currently, no approved pharmacologic therapies specifically target Lp(a) reduction. Some lipid-lowering agents, *e.g.* proprotein convertase subtilisin/kexin 9 inhibitors and cholesteryl ester transfer protein inhibitors, demonstrate only modest Lp(a) lowering effects (*1*).

More than 90% of the variation in Lp(a) concentrations in blood is determined by genetic factors, with significant differences observed between ethnic groups and among European populations (*3*, *4*). Studies suggest that Lp(a) concentrations may increase with age, particularly in postmenopausal women (*5*, *6*). Additionally, various non-genetic factors, including kidney, liver, and thyroid diseases, inflammation and certain medications, can influence Lp(a) concentrations. Accordingly, current guidelines recommend interpreting Lp(a) values with caution in individuals with these conditions (*1*, *2*).

To date, only a few studies have examined the distribution of Lp(a) values in the Polish adult population (*7*-*9*). However, data on Lp(a) distribution in Polish children are still lacking. Beyond the adult population, there is growing recognition of the need to assess Lp(a) concentrations in children. Pediatric guidelines recommend lipid screening at two key time points, *i.e.,* between 9-11 and 17-21 years, to identify individuals at risk for early-onset atherosclerotic cardiovascular disease (ASCVD) (*10*). Incorporating Lp(a) measurements into pediatric ASCVD risk assessment is also desirable (*11*). Nevertheless, no study has evaluated Lp(a) concentrations in Polish adults and children at the same time. Therefore, this study aims to assess Lp(a) concentrations in the Polish population, encompassing both adults and children, while considering age, sex, and selected non-genetic factors. It also examines the prevalence of elevated Lp(a) values and the association with cardiometabolic risk factors.

## Materials and methods

### Study design

**Figure 1:**
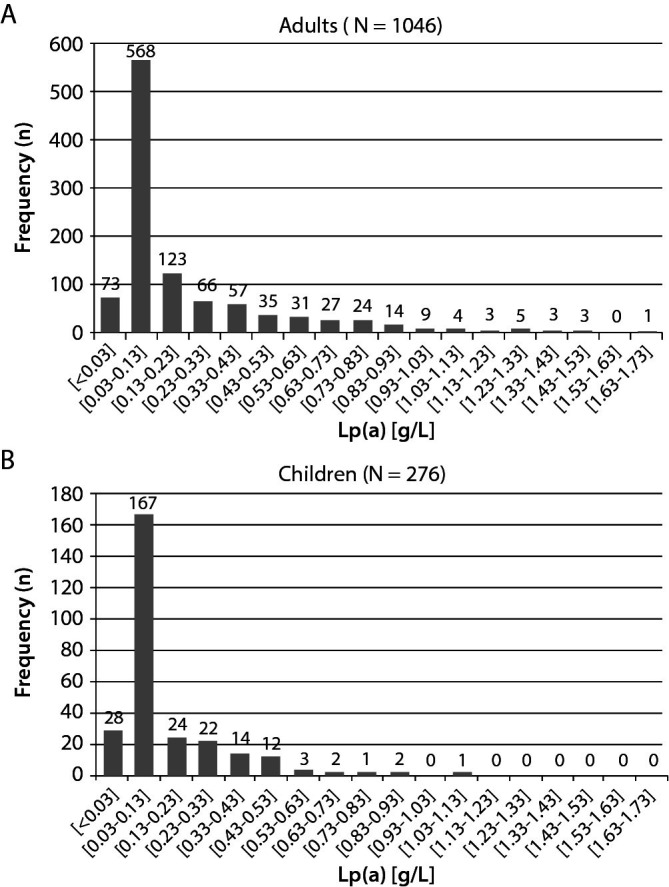
Distribution of serum Lp(a) concentrations in adults (A) and children (B). Lp(a) - lipoprotein(a).

This cross-sectional study included a total number of 1322 presumably healthy Polish subjects, selected based on defined inclusion and exclusion criteria (Figure 1S, supplementary data). Between 2011 and 2019, adults were recruited through workplaces, website announcements, and wall posters in public places, while children were recruited from primary schools. All individuals (parents for children) provided written informed consent. The study was conducted in accordance with the Declaration of Helsinki and approved by the Bioethics Committee of Nicolaus Copernicus University in Toruń, Collegium Medicum in Bydgoszcz, Poland (approval no. KB 507/2024, 10.12.2024).

Exclusion criteria included pregnancy, growth hormone replacement therapy, a history of chronic or acute inflammation or serum C-reactive protein (CRP) concentrations ≥ 10 mg/L, an estimated glomerular filtration rate (eGFR) ≤ 60 mL/min/1.73 m^2^, and thyroid disorders. Thyroid-related exclusion criteria encompassed thyrostatic treatment, radioactive iodine therapy, thyroid hormone replacement therapy, or thyroid-stimulating hormone (TSH) concentrations in serum outside the previously published reference ranges (*12*, *13*). Individuals with serum LDL-cholesterol (LDL-C) concentrations ≥ 4.91 mmol/L (≥ 190 mg/dL), as well as with chronic liver disease or alanine aminotransferase (ALT) activity in serum exceeding two times the sex-specific upper reference limits: 0.82 µkat/L (49 U/L) for men and 0.55 µkat/L (33 U/L) for women were also excluded.

### Definition of subject characteristics and cardiometabolic parameters

Subject characteristics were determined based on demographic, clinical and laboratory parameters. Age, smoking habits, medical history and medication use were collected through a medical questionnaire, conducted by trained medical staff: general practitioners and laboratory specialists. Height (cm) and weight (kg) were measured using standard methods, and body mass index (BMI) derived. In adult, overweight was defined as a BMI between 25 and 29.9 kg/m^2^, while obesity was classified as a BMI of ≥ 30 kg/m^2^. In children, overweight was defined as BMI ranging from 85th to less than the 95th percentile, while obesity was defined as BMI ≥ 95th percentile, based on the Centers for Disease Control and Prevention’s sex-specific BMI-for-age growth charts (*14*). Systolic and diastolic blood pressure were measured twice in a seated position after at least 10 min of rest, following standard procedures. Hypertension was defined in adults as blood pressure ≥ 140/90 mmHg or use of antihypertensive treatment, and in children as blood pressure ≥ 95^th^ percentile, based on the sex and height-specific charts from the Polish Hypertension Society recommendations (*15*). Smoking status was categorized as: current smoker, ex-smoker, or non-smoker. Dyslipidemia in adults was characterized by at least one of the following findings: serum triglycerides (TG) ≥ 1.7 mmol/L (≥ 150 mg/dL), total cholesterol (TC) ≥ 5.0 mmol/L (≥ 190 mg/dL), LDL-C ≥ 3.0 mmol/L (≥ 115 mg/dL), non-high density lipoprotein (HDL) cholesterol (non-HDL-C) ≥ 3.4 mmol/L (≥130 mg/dL), apolipoprotein B (apoB) > 1.00 g/L, HDL-cholesterol (HDL-C) ≤ 1.2 mmol/L (≤ 45 mg/dL) for women and ≤ 1.0 mmol/L (≤ 40 mg/dL) for men, respectively, or use of lipid-lowering medications (*2*). In children, dyslipidemia was defined by at least one abnormal serum lipid parameter as follows: TC ≥ 4.4 mmol/L (≥ 170 mg/dL), TG ≥ 0.85 mmol/L (≥ 75 mg/dL) for children < 10 years old and ≥ 1.02 mmol/L (≥ 90 mg/dL) for those aged 10-11 years, respectively; LDL-C ≥ 2.85 mmol/L (≥ 110 mg/dL), HDL-C ≤ 1.17 mmol/L (≤ 45 mg/dL), and non-HDL-C ≥ 3.1 mmol/L (≥ 120 mg/dL) (*2*). Impaired fasting glucose and type 2 diabetes were diagnosed according to the American Diabetes Association criteria (*16*). Serum CRP concentrations of 3.0 to 9.9 mg/L were considered as low-grade inflammation. A postmenopausal status was assigned to women aged ≥ 52 years (*17*). Cardiovascular risk thresholds for Lp(a) were set at < 0.30 g/L (< 75 nmol/L) for low, 0.30-0.50 g/L (75-125 nmol/L) for intermediate, and > 0.50 g/L (> 125 nmol/L) for high/very high risk (*2*). The study considered hypertension, prediabetes/diabetes, smoking, dyslipidemia, low-grade inflammation, overweight/obesity, and postmenopausal status as cardiometabolic risk factors.

### Laboratory measurements

Fasting venous blood samples were collected from each participant at 7.00-9.00 a.m. using disposable Vacutainer system (Becton Dickinson, Franklin Lakes, USA) into a 6-mL tube with clot activator to obtain serum for biochemical measurements, two 2-mL tubes containing EDTA for HbA_1c_ and sodium fluoride for glucose measurements, respectively. Samples with clot activator were left for 30 minutes at room temperature to clot completely and then centrifuged at 3000 rpm for 15 minutes. Samples for glucose testing were centrifuged immediately after collection at 3000 rpm for 15 minutes at 4 °C. Concentrations of plasma glucose and HbA_1c_ in the whole blood were measured in fresh samples, while TC, HDL-C, TG, apoB, Lp(a), creatinine, CRP, TSH, ALT, aspartate aminotransferase (AST), and γ-glutamyltransferase (GGT) were assayed in deep-frozen (− 80 °C) serum samples. Samples were stored for a maximum of 2 years.

All basic biochemical measurements were performed on the Alinity platform (Abbott Laboratories, Chicago, USA). Reagents for measuring apoB and Lp(a), using immunoturbidimetric methods, were supplied by Randox Laboratories Ltd. (Crumlin, Northern Ireland, UK). Lipoprotein(a) and apoB measurements were performed using the ABX Pentra 400 analyzer (Horiba ABX, Montpellier, France). The apoB assay is traceable to the IFCC/WHO reference material SP3-08. The Lp(a) assay, licensed from Denka Seiken, is based on an antigen-antibody reaction between Lp(a) in a sample and anti-Lp(a) (rabbit) polyclonal antibodies adsorbed to latex particles. This assay utilizes a five-point calibration (analytical measurement range 0.03-0.90 g/L) and is traceable to the WHO/IFCC reference material SRM 2B. Lipoprotein(a) measurements showed a between-run imprecision (as coefficient of variation, CV) < 6.1%, with a limit of detection (LoD) of 0.03 g/L. Low density lipoprotein-C was calculated using the Sampson formula, while non-HDL-C was determined by subtracting HDL-C from TC (*18*). An estimated glomerular filtration rate was assessed using the 2021 CKD-EPI equation for adults and the Schwartz formula for children, based on serum creatinine concentration measured by the enzymatic method. Non-Lp(a) apoB was calculated after converting Lp(a) and apoB concentrations from g/L to nmol/L. The conversion factors used were 0.00055 for apoB from nmol/L to g/L, and 1818 for apoB from g/L to nmol/L. For Lp(a), to convert g/L to nmol/L results were multiplied by 215 (*19*). The number of non-Lp(a) apoB-containing particles was determined by subtracting Lp(a) particle count (in nmol/L) from the total apoB particle count (in nmol/L) and then converting this value to g/L by multiplying by 0.00055. Lipoprotein(a)-cholesterol (Lp(a)-C) was estimated using the Rosenson-Marcovina formula as: Lp(a) (nmol/L) x 0.077 = Lp(a)-C (mg/dL). The calculated Lp(a)-C was then subtracted from LDL-C to obtain LDL-C_Lp(a)corr_ (*20*). This formula was selected because studies on large cohorts have shown that the conventional method, which assumes Lp(a)-C comprising 30% of Lp(a) mass, overestimates Lp(a)-C at higher Lp(a) concentrations, leading to an underestimation of LDL-C_Lp(a)corr_. The Rosenson-Marcovina formula estimates Lp(a)-C as 16.6% of Lp(a) mass, providing a more accurate correction.

### Statistical analysis

Statistical analysis was performed using Statistica 13.3 software (StatSoft Inc., Tulsa, USA). Normality was assessed using the Shapiro-Wilk test and data presented as median with Q1-Q3 ranges (25^th^-75th percentile) for non-Gaussian distributions. Lipoprotein(a) values < LoD (0.03 g/L) were recorded as 0.03 for calculations. Comparisons between two groups were performed using the Student’s t-test (Gaussian) or the Mann–Whitney U test (non-Gaussian data distribution), while multiple-group comparisons were performed using the Kruskal–Wallis test. Fisher’s exact test or the Chi-squared test were used to compare categorical variables. Two-way ANOVA models were used to evaluate median values and 95% confidence interval (CI) for medians by increasing age. Statistical significance (P) for age by sex interaction was calculated with tests of between-subject effects. Spearman rank correlation coefficients were calculated between Lp(a) and cardiometabolic risk factors. Multivariable linear regression was used to assess the relationship between Lp(a) (dependent variable) and cardiometabolic risk factors (independent variables), with adjusted R^2^ reported. Logistic regression examined associations between high Lp(a) concentrations and cardiometabolic risk factors, with odds ratios calculated *per* unit increase in risk factor occurrence. For multivariable analyses, non-Gaussian variables were log-transformed. A P-value < 0.05 was considered statistically significant.

## Results

### General characteristics of studied population and lipid profile analysis

**Figure 2:**
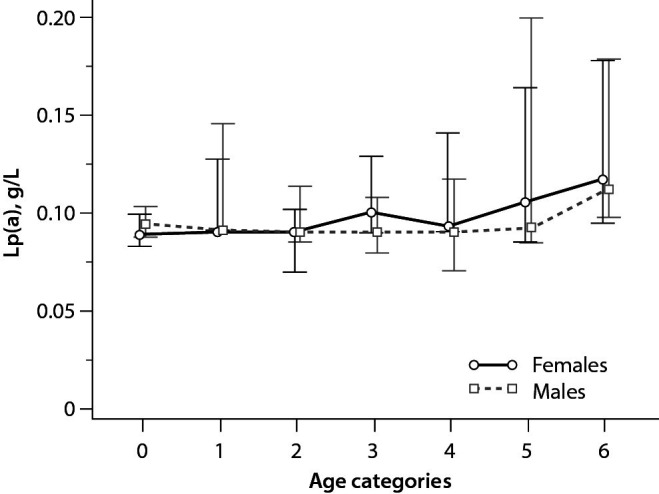
Serum lipoprotein(a) concentrations across age categories. Data are expressed as medians, with vertical bars indicating 95% confidence intervals. Age categories (x-axis): 0: 9-11 years, 1: 18-29 years, 2: 30-39 years, 3: 40-49 years, 4: 50-59 years, 5: 60-69 years, 6: 70-99 years. Lp(a) - lipoprotein(a).

The study included 1046 Caucasian adults (54% women), aged 18-99 years, and 276 prepubertal Caucasian children (aged 9-11 years). Participants originated from both urban and rural areas of the Kuyavian-Pomeranian Voivodeship, a region in north-central Poland (Figure 2S, supplementary data). Table 1 summarizes the characteristics of studied subjects. In adults, the prevalence of overweight and dyslipidemia, characterized by elevated LDL-C, TG, apoB, non-HDL-C, and/or lower HDL-C, was significantly higher in men than in women. Among children, overweight was significantly more frequent in boys than in girls (16.4% *vs.* 8.1%; P = 0.034). The prevalence of hypertension was almost 3-times higher in girls than in boys, even if with a borderline statistical significance (P = 0.055).

**Table 1:** Characteristics of recruited subjects

	**Total adults** **(N = 1046)**	**Adult women** **(N = 562)**	**Adult men** **(N = 484)**	**P-value women *vs*. men**	**Total children** **(N = 276)**	**Girls** **(N = 148)**	**Boys** **(N = 128)**	**P-value girls *vs*. boys**
Age, years*	42 (18-99)	42 (33-56)	42 (32-54)	0.230	10 (9-11)	10 (9-11)	10 (9-11)	0.522
Menopause	-	32.7%	-	-	-	-	-	-
**Risk factors**								
Overweight	34.4%	26.2%	44.0%	< 0.001	12.0%	8.1%	16.4%	0.034
Obesity	16.0%	15.2%	17.2%	0.381	12.3%	10.1%	14.8%	0.236
CRP 3-9.9 mg/L	13.6%	15.0%	11.9%	0.144	9.1%	9.5%	8.6%	0.795
Prediabetes	19.8%	17.9%	21.9%	0.105	11.4%	10.2%	12.8%	0.499
Type 2 diabetes	5.6%	4.9%	6.3%	0.324	0	0	0	-
TC ≥ 5.0 mmol/L (adults), ≥ 4.4 mmol/L (children)	56.2%	57.1%	55.2%	0.537	50.7%	52.7%	48.4%	0.477
LDL-C ≥ 3.0 mmol/L (adults), ≥ 2.85 mmol/L (children)	52.1%	48.9%	55.8%	0.026	28.3%	29.1%	27.3%	0.741
ApoB > 1.00 g/L	36.5%	49.1%	58.1%	0.004	12.3%	13.5%	10.9%	0.513
TG ≥ 1.7 mmol/L (adults), ≥ 0.85/≥ 1.02 mmol/L (children)	19.6%	12.8%	27.5%	<0.001	33.3%	31.8%	35.2%	0.513
HDL-C ≤ 1.2/≤ 1.0 mmol/L (women/men), ≤ 1.17 mmol/L (children)	13.0%	7.1%	19.9%	<0.001	10.5%	10.8%	10.2%	0.872
Non-HDL-C ≥ 3.4 mmol/L (adults), ≥ 3.1 mmol/L (children)	58.8%	52.9%	65.7%	<0.001	37.3%	39.2%	35.2%	0.494
eGFR 60-89 mL/min/1.73m^2^	24.9%	24.6%	25.3%	0.794	3.3%	3.1%	3.5%	0.853
Hypertension	22.3%	21.1%	23.7%	0.314	6.2%	8.8%	3.2%	0.055
Current smoker	19.3%	19.9%	18.5%	0.567	0	0	0	-
Ex-smokers	16.0%	11.0%	21.0%	<0.001	0	0	0	-
*Median (min-max). ApoB - apolipoprotein B. CRP - C-reactive protein. eGRF - estimated glomerular filtration rate. HDL-C - high-density lipoprotein cholesterol. LDL-C - low-density lipoprotein cholesterol. non-HDL-C - non-high density lipoprotein cholesterol. TC - total cholesterol. TG - triglycerides.								

Adults had significantly higher concentrations of TC, TG, non-HDL-C, LDL-C, and apoB, and lower HDL-C concentrations than children, with similar differences observed between women and girls, and men and boys (Table 2). While Lp(a) concentrations did not differ by sex, they were significantly higher in adults than children, and in women than girls, a pattern also seen for Lp(a)-C. The percentage contributions of Lp(a)-apoB to total apoB (%Lp(a)/apoB) and Lp(a)-C to LDL-C (%Lp(a)-C/LDL-C) were significantly higher in boys when compared to girls, with simultaneously lower values of the percentage contribution of Lp(a)-apoB to total apoB in boys compared to men. Moreover, non-Lp(a) apoB and LDL-C_Lp(a)corr_ were significantly higher in adults than in children in both sexes.

**Table 2:** Lipid parameters in studied subjects.

**Parameter**	**All adults** **(N = 1046)**	**Adult women** **(N = 562)**	**Adult men (N = 484)**	**Women *vs.* men**	**All children (N = 276)**	**Girls (N = 148)**	**Boys (N = 128)**	**Girls *vs.*** **boys**	**Adults *vs.* children**	**Women *vs.* girls**	**Men *vs.* boys**
TC, mmol/L	5.04 (4.42-5.74)	5.04 (4.45-5.69)	4.99 (4.40-5.79)	0.482	4.40 (3.85-4.86)	4.45 (3.93-4.84)	4.34 (3.80-4.91)	0.612	< 0.001	< 0.001	< 0.001
TG, mmol/L	1.09 (0.79-1.52)	0.95 (0.71-1.31)	1.26 (0.96-1.78)	< 0.001	0.79 (0.60-1.08)	0.82 (0.62-1.02)	0.73 (0.56-1.09)	0.240	< 0.001	< 0.001	< 0.001
HDL-C, mmol/L	1.42 (1.21-1.68)	1.58 (1.37-1.86)	1.27 (1.09-1.47)	< 0.001	1.50 (1.32-1.73)	1.52 (1.32-1.71)	1.47 (1.29-1.76)	0.551	0.001	0.028	< 0.001
Non-HDL-C, mmol/L	3.54 (2.92-4.29)	3.39 (2.84-4.16)	3.77 (3.08-4.45)	< 0.001	2.82 (2.38-3.28)	2.89 (2.43-3.31)	2.74 (2.35-3.26)	0.595	< 0.001	< 0.001	< 0.001
LDL-C, mmol/L	3.05 (2.46-3.72)	2.95 (2.40-3.65)	3.15 (2.53-3.77)	0.022	2.46 (2.02-2.90)	2.48 (1.99-2.90)	2.40 (1.99-2.87)	0.613	< 0.001	< 0.001	< 0.001
ApoB, g/L	0.90 (0.76-1.07)	0.85 (0.72-1.02)	0.97 (0.80-1.14)	< 0.001	0.77 (0.65-0.92)	0.78 (0.65-0.92)	0.77 (0.64-0.89)	0.406	< 0.001	< 0.001	< 0.001
Lp(a), g/L nmol/L*	0.09 (0.06-0.26) 19.3 (12.9-55.9)	0.10 (0.06-0.27) 21.5 (12.9-58.0)	0.09 (0.06-0.26) 19.3 (12.9-55.9)	0.465	0.09 (0.05-0.15) 19.3 (10.7-32.2)	0.09 (0.05-0.13) 19.3 (10.7-27.9)	0.09 (0.06-0.24) 19.3 (12.9-51.6)	0.155	0.014	0.003	0.708
Lp(a) (%): > 0.30 g/L 0.30-0.50 g/L > 0.50 g/L	22 9 13	22 9 13	22 10 12	1.000 0.582 0.626	18 13 5	15 11 4	22 16 6	0.133 0.223 0.444	0.148 0.047 < 0.001	0.061 0.459 0.002	1.000 0.056 0.050
Non-Lp(a) apoB, g/L	0.87 (0.73-1.05)	0.83 (0.70-0.99)	0.95 (0.78-1.11)	< 0.001	0.77 (0.64-0.89)	0.77 (0.64-0.90)	0.75 (0.63-0.86)	0.276	< 0.001	< 0.001	< 0.001
%Lp(a)/apoB	1.39 (0.74-3.34)	1.46 (0.78-3.46)	1.31 (0.70-3.24)	0.055	1.74 (0.76-3.10)	1.36 (0.71-2.32)	1.67 (1.00-4.09)	0.034	0.947	0.069	0.044
Lp(a)-C, mg/dL	1.53 (0.99- 4.33)	1.61 (0.99-4.42)	1.49 (0.99- 4.29)	0.466	1.50 (0.74-2.48)	1.47 (0.74-2.11)	1.56 (1.04-3.96)	0.051	0.014	0.003	0.708
LDL-C_Lp(a)corr_, mg/dL	113 (92-139)	110 (91-136)	118 (95-143)	0.015	91 (76-109)	94 (77-110)	90 (75-107)	0.708	< 0.001	< 0.001	< 0.001
%Lp(a)-C/LDL-C	1.53 (0.81-3.69)	1.59 (0.84-3.69)	1.43 (0.79-3.80)	0.952	1.70 (0.91-3.08)	1.69 (0.82-2.60)	1.75 (1.12-3.71)	< 0.001	0.711	0.371	0.127
Data are expressed as medians (25^th^ – 75^th^ percentile); *conversion factor from g/L to nmol/L: 215. ApoB - apolipoprotein B. HDL-C - high-density lipoprotein cholesterol. LDL-C - low-density lipoprotein cholesterol. Lp(a) - lipoprotein(a). Lp(a)-C - lipoprotein(a) cholesterol. %Lp(a)/apoB - contribution of Lp(a)-apoB to total apoB in %. %Lp(a)-C/LDL-C - contribution of Lp(a)-C to LDL-C in %. non-HDL-C - non-high density lipoprotein cholesterol. TC - total cholesterol. TG - triglycerides.											

### Distribution of Lp(a) across age categories

Figure 1 illustrates the distribution of Lp(a) concentrations in serum of adults and children in the studied population. Lipoprotein(a) concentrations were undetectable (*i.e.,* < 0.03 g/L) in 7% of adults and 10% of children. Lipoprotein(a) > 0.30 g/L was observed in 22% of adults and 18% of children, with no significant sex difference. However, Lp(a) > 0.50 g/L was significantly more frequent in adults (13%) than children (5%) (P < 0.001) (Table 2).

**Figure 3:**
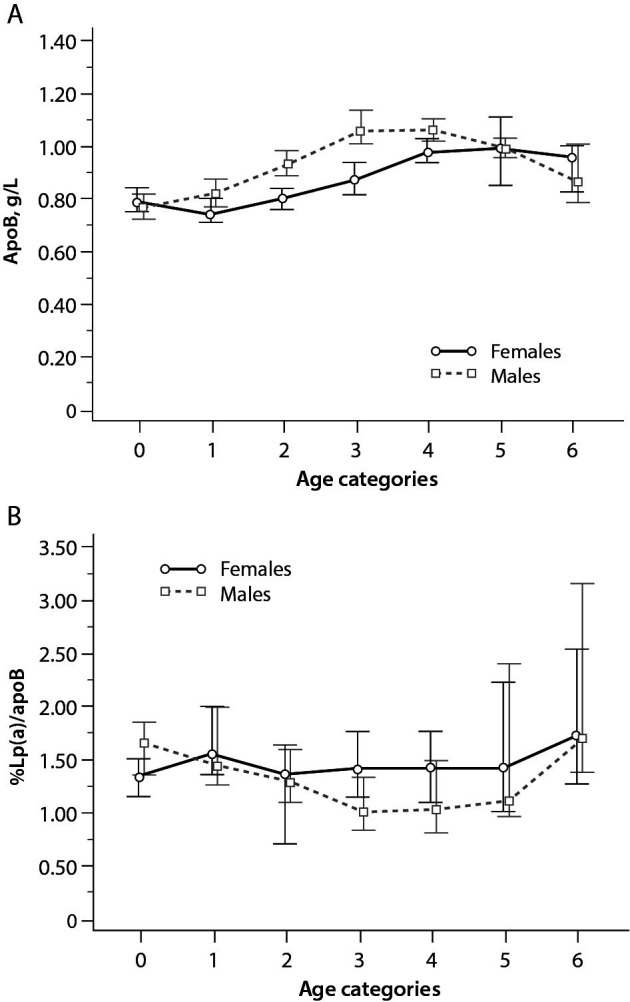
Serum apoB concentrations (A) and %Lp(a)/apoB values (B) across age categories. Data are expressed as medians, with vertical bars indicating 95% confidence intervals. Age categories (x-axis): 0: 9-11 years, 1: 18-29 years, 2: 30-39 years, 3: 40-49 years, 4: 50-59 years, 5: 60-69 years, 6: 70-99 years. ApoB - apolipoprotein B. %Lp(a)/apoB - contribution of Lp(a)-apoB to total apoB in %.

**Figure 4:**
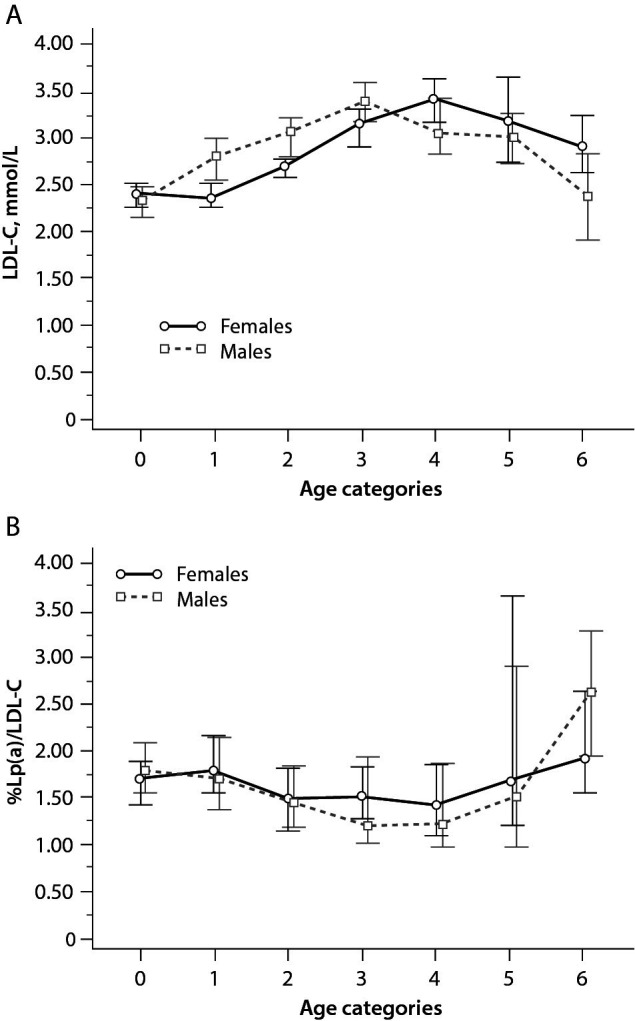
Changes in serum LDL-C (A) and %Lp(a)-C/LDL-C values (B) across age categories. Data are expressed as medians, with vertical bars indicating 95% confidence intervals. Age categories (x-axis): 0: 9-11 years, 1: 18-29 years, 2: 30-39 years, 3: 40-49 years, 4: 50-59 years, 5: 60-69 years, 6: 70-99 years. LDL-C - low-density lipoprotein cholesterol. %Lp(a)-C/LDL-C - contribution of Lp(a)-C to LDL-C in %.

Figure 2 depicts the association of Lp(a) concentrations with age and sex in the studied population. Lipoprotein(a) concentrations were significantly higher only in females after the age of 50 (P for trend, 0.0008). On the contrary, the slight increase of Lp(a) concentrations in males after the age of 60 was not significant (P for trend, 0.157). Similar models were applied to evaluate the association of apoB concentrations in serum and the contribution of Lp(a)-apoB to total apoB (%Lp(a)/apoB) with age and sex (Figure 3). In men, apoB concentrations were significantly higher, reaching a plateau in the fourth decade, then decreasing after age 50 (P for trend, < 0.0001). In women, the increase was late, with a plateau around the fifth decade (P for trend, < 0.0001). The contribution of Lp(a)-apoB to total apoB did not change significantly with age in women (P = 0.064), whereas in men there were lower values until the fourth decade and then an increase in later years (P = 0.002). The dynamics of apoB changes and the percentage contribution of Lp(a)-apoB to apoB differed significantly between males and females (P for apoB, < 0.0001, P for %Lp(a)/apoB, 0.044). By analyzing LDL-C and the contribution of Lp(a)-C to LDL-C (Figure 4), we found that LDL-C concentrations were significantly higher until the fourth decade of life in males and the fifth decade in females, followed by a gradual decline in later years (P for trend, < 0.0001). The dynamics of LDL-C changes differed significantly between males and females (P < 0.0001). In males, the percentage contribution of Lp(a)-C to LDL-C was slightly lower until the fourth decade and then gradually higher in later years (P for trend, 0.0018). In females, %Lp(a)-C/LDL-C changes were less evident, with a modest significant increase after the fifth decade (P for trend, 0.047). The dynamics of %Lp(a)-C/LDL-C changes did not differ significantly between sexes (P = 0.345).

### Correlations between Lp(a) and cardiometabolic parameters, and multivariable logistic regression models assessing independent predictors of high Lp(a) concentrations

In adults, no correlations between Lp(a) concentration and cardiometabolic parameters exceeded r = 0.25. In children, Lp(a) showed weak correlations with non-HDL-C (r = 0.26, P < 0.001) and LDL-C (r = 0.27, P < 0.001).

A multivariable logistic regression model was used to assess independent predictors of high Lp(a) concentrations (≥ 0.30 g/L; Table 3). Among the analyzed variables, only increased LDL-C remained a significant predictor both in women and children, with an approximately two-fold increased likelihood of elevated Lp(a) in adult women (odds ratio, OR = 1.77; 95%CI: 1.10-2.86; P = 0.021) and tripling the probability of the occurrence of high Lp(a) concentrations in children (OR = 2.83; 95%CI: 1.24-6.44; P = 0.009). Additionally, boys had an approximately twofold higher probability of Lp(a) concentrations ≥ 0.30 g/L than girls (OR = 2.17; 95%CI: 1.05-4.46; P = 0.024).

**Table 3:** Predictors of high Lp(a) concentrations in serum by the multivariable logistic regression model.

	**All adults**		**Adult women**		**Adult men**		**Children**
Lp(a), g/L	≥ 0.30	≥ 0.50	≥ 0.30	≥ 0.50	≥ 0.30	≥ 0.50	≥ 0.30
Overall model statistics	Chi^2^=10.9 P=0.143 NR^2^=0.017	Chi^2^=10.8 P=0.148 NR^2^=0.021	Chi^2^=8.5 P=0.204 NR^2^=0.024	Chi^2^=12.1 P=0.055 NR^2^=0.043	Chi^2^=4.53 P=0.606 NR^2^=0.015	Chi^2^=4.19 P=0.651 NR^2^=0.019	Chi^2^=16.4 P=0.012 NR^2^=0.110
	Odds ratio (95% confidence intervals)						
LDL-C, ≥ 3.0 mmol/L (adults) ≥ 2.85 mmol/L (children)	1.51 (1.05-2.16)	1.42 (0.91-2.23)	**1.77** **(1.10-2.86)**	**2.03** **(1.11-3.69)**	1.15 (0.66-2.00)	0.79 (0.38-1.63)	**2.83** **(1.24-6.44)**
ApoB, ≥ 1.0 g/L	1.08 (0.74-1.56)	1.28 (0.81-2.04)	0.99 (0.59-1.67)	1.06 (0.57-1.97)	1.32 (0.76-2.28)	1.75 (0.85-3.60)	1.45 (0.52-4.08)
eGFR, 60-89 mL/min/1.73 m^2^	0.99 (0.98-1.00)	0.99 (0.98-1.01)	1.28 (0.75-2.17)	1.23 (0.66-2.31)	1.28 (0.74-2.21)	1.04 (0.51-2.14)	1.00 (0.99-1.01)
Low-grade inflammation (CRP ≥ 3.0 mg/L)	1.05 (0.67-1.66)	1.10 (0.64-1.89)	0.87 (0.46-1.61)	1.21 (0.60-2.44)	1.32 (0.67-2.58)	1.07 (0.44-2.61)	1.75 (0.52-5.94)
BMI, ≥ 30 kg/m^2^ (adults) > 95th percentile (children)	1.02 (0.66-1.58)	1.32 (0.80-2.17)	1.22 (0.66-2.27)	1.13 (0.55-2.31)	0.87 (0.47-1.63)	1.42 (0.70-2.90)	0.37 (0.10-1.40)
Menopause	-	-	0.86 (0.50-1.46)	1.22 (0.65-2.29)	-	-	-
Age ≥ 50 years	0.84 (0.57-1.23)	1.03 (0.64-1.64)	-	-	0.78 (0.46-1.33)	0.71 (0.361.40)	-
Sex (male)	0.94 (0.69-1.29)	0.86 (0.58-1.27)	-	-	-	-	**2.17** **(1.05-4.46)**
Values in bold denote statistical significance of odds ratio (P < 0.05). ApoB - apolipoprotein B. BMI - body mass index. CRP - C-reactive protein. eGFR - estimated glomerular filtration rate. LDL-C - low-density lipoprotein cholesterol. Lp(a) - lipoprotein(a). NR^2^ - Nagelkerke R^2^.							

## Discussion

Understanding the distribution of Lp(a) concentrations in relation to age, sex, and cardiometabolic risk factors may allow for a more accurate assessment of cardiovascular risk. Given that Lp(a) contributes to both apoB and LDL-C concentrations, its impact should be considered in routine lipid profiling to avoid potential misclassification of risk (*1*, *2*).

Our findings indicate that approximately one-fifth of both adults and children had Lp(a) ≥ 0.30 g/L. However, Lp(a) > 0.50 g/L was significantly more prevalent in adults than in children. While no significant sex differences were observed, median Lp(a) concentrations tended to be lower in girls. Interestingly, Lp(a) concentrations were significantly higher in women after the age of 40, whereas in men only a slight rise was observed after age of 60. Despite these variations, the overall age-related dynamics of Lp(a) were similar in both sexes. In contrast, the analysis of %Lp(a)/apoB and %Lp(a)-C/LDL-C ratios showed lower values in men up to their 40s and higher values in both sexes after the age of 50, indicating that the contribution of Lp(a) to apoB and LDL-C measurements is influenced by both age and sex. Several studies have reported higher Lp(a) concentrations in women. For example, the MESA study demonstrated that women consistently had higher Lp(a) concentrations across all ethnic groups (*21*). Other studies have indicated that the prevalence of Lp(a) > 0.30 g/L varies depending on the presence of dyslipidemia, with higher concentrations observed in women than in men (*22*). A recent study by Yurtseven *et al.* confirmed that Lp(a) concentrations were significantly higher in women over 50 years of age, suggesting the need for sex-specific Lp(a) thresholds in cardiovascular risk assessment (*23*). Data from the Copenhagen General Population Study showed that Lp(a) concentrations increased around age 50 in women, irrespective of use of hormone replacement therapy (*5*). However, when examining the association between Lp(a) > 0.40 g/L and CVD mortality, no significant sex differences were noted. In contrast, the Lp(a)HERITAGE study demonstrated that individuals < 65 years old, particularly women, tend to have higher Lp(a) and LDL-C concentrations, potentially increasing their risk of premature CVD (*24*). Evidence suggests that sex differences in lipoprotein production result from a combination of genetic factors, sex chromosomes, and sex hormones. Lipoprotein(a) production is highly regulated by the *LPA* gene, with variations in its expression and in the activity of proteins, such as sortilin, involved in Lp(a) production and clearance, potentially contributing to higher Lp(a) concentrations in women compared to men (*21*). However, the exact mechanisms behind these differences remain unclear.

Few studies have examined Lp(a) concentrations in Polish population. The EUROASPIRE V survey, conducted on 200 subjects without diagnosed CVD, found that 30% of participants had Lp(a) concentration > 0.30 g/L, and 22.5% had Lp(a) > 0.50 g/L (*7*). In the Malopolska CAD Prophylactic Program, enrolling 800 middle-aged individuals, 17.8% of participants had Lp(a) > 0.50 g/L, with a higher proportion of women in this group (*8*). The STAR-Lp(a) study, including 2475 outpatient cardiology clinic patients, reported that Lp(a) ≥ 0.30 g/L was present in 21.5% of participants, while Lp(a) ≥ 0.50 g/L was found in 13.5% of individuals (*9*). However, none of these studies analyzed Lp(a) variations across different age groups. In a previous study, we examined the association between Lp(a) and lipid biomarkers of cardiovascular risk in relation to age and sex in 422 apparently healthy individuals aged 19-84 years (*25*). We found that women aged ≤ 40 years with LDL-C ≤ 100 mg/dL and elevated Lp(a) (≥ 0.40 g/L) had higher apoB and small dense LDL-C concentrations compared to those with Lp(a) < 0.40 g/L. This pattern was not observed in men. These findings suggested that younger women with elevated Lp(a) may have an increased CVD risk related to higher apoB concentrations, despite having desirable LDL-C concentrations.

Data on Lp(a) in Polish children are very limited. Research in pediatric endocrinology suggested that children with type 1 diabetes have significantly higher Lp(a) concentrations than healthy controls (*26*). Additionally, Lp(a) was linked to a family history of CVD in Polish children with diabetes, obesity, and hypertension (*27*). In young Finns, Raitakari *et al.* found an increase in Lp(a) concentrations and prevalence of elevated Lp(a) (> 0.30 g/L) with age (*11*). Elevated Lp(a) in youth, particularly when combined with high LDL-C, was associated with a significantly higher CVD risk. Finally, the prospective “LIFE Child” study, performed in a German children cohort, found that Lp(a) concentrations remained stable over 4 years, unaffected by age, sex, or BMI, but strongly associated with LDL-C (*28*).

To our knowledge, the present study is the first evaluating the Lp(a)-dependent lipid biomarkers in the Polish population, considering both sex and age. It should be noted that total apoB measurements primarily reflect the sum of LDL-apoB and Lp(a)-apoB, while LDL-C measurements can be influenced by the cholesterol content of Lp(a) (Lp(a)-C). Recent studies have suggested that Lp(a)-apoB is more strongly associated with ASCVD risk than LDL-apoB (*29*). The proatherogenic properties of Lp(a) arise from its apoB and cholesterol content, oxidized phospholipids, proinflammatory and antifibrinolytic characteristics, and denser structure compared to LDL particles. However, the overall contribution of Lp(a) to CVD risk is smaller than that of LDL particles, particularly in individuals with low Lp(a) concentrations (*30*). In our study, the median Lp(a)/apoB was 1.39% in adults and 1.74% in children, respectively. A UK Biobank study reported a median of 0.9% in primary prevention subjects (*29*). Furthermore, the median Lp(a)-C/LDL-C in our study was 1.53% in adults and 1.70% in children, ~5% lower than that reported in a meta-analysis of 18,043 individuals, which however used an overestimating Lp(a)-C calculation. We applied the Rosenson-Marcovina formula (considering Lp(a)-C as 16.6% of Lp(a) mass) for our exploratory analysis, though it requires further validation. Given such inaccuracies, currently LDL-C correction based on Lp(a)-C is not recommend.

Ratio %Lp(a)/apoB remained stable with age in women but declined in men until the fourth decade before increasing significantly. Ratio %Lp(a)-C/LDL-C was lower in men until the fourth decade, then increasing, as in women, after 50 years of age. This trend coincided with decreasing apoB and LDL-C concentrations and rising of Lp(a), suggesting greater lipid profile atherogenicity in older men. Interestingly, in our population the highest %Lp(a)/apoB and %Lp(a)-C/LDL-C were seen in boys aged 9-11, despite relatively low LDL-C and apoB concentrations. As no previous studies have assessed these relationships, our results cannot be directly compared with those of other authors.

The variability in Lp(a) concentrations is primarily determined by genetic factors. However, recent studies suggest that Lp(a) is also interrelated with other cardiovascular risk factors, such as apoB, CRP, and oxidized phospholipids, which may influence cardiovascular risk assessment (*31*, *32*). In our study, weak correlations between Lp(a) and LDL-C or non-HDL-C concentrations were observed only in children. Moreover, an LDL-C concentration ≥ 110 mg/dL was the strongest predictor of high Lp(a) concentrations, increasing approximately three-fold the likelihood to detect this condition. Additionally, in women, an LDL-C concentration ≥ 115 mg/dL was a significant predictor of high Lp(a) concentrations, approximately doubling the likelihood of detecting elevated Lp(a). These findings align with our previous observations that LDL-C is a stronger predictor of high Lp(a) concentrations in women than in men (*25*). Similarly, Burzyńska *et al.* reported that individuals with elevated Lp(a) were more likely to be females having hyperlipidemia (*9*).

The findings of our study are especially relevant considering that Poland is classified as a high-risk country for cardiovascular mortality and shows a relatively high prevalence of elevated Lp(a) concentrations. Moreover, atherogenic isoforms of apo(a), characterized by smaller apo(a) size, fewer KIV-2 repeats, and the presence of the rs10455872 variant, are more frequently observed in Central and Western Europe (*e.g.*, Poland, Germany, the United Kingdom) as well as in the Balkan region (Southeastern Europe, *e.g*., Bulgaria, Romania), compared to Northern and Southern Europe (*e.g*., Finland, Italy, Greece, Spain). Therefore, Lp(a) characteristics could be region-specific (*8*, *33*).

Some study strengths and limitations should be further acknowledged. To our knowledge, it is the first study to evaluate Lp(a)-dependent lipid biomarkers in the Polish population while considering both sex and age. Although multiple studies have explored the distribution of Lp(a) concentrations in relation to cardiometabolic risk factors, comparisons are often difficult due to ethnic variability and confounding factors affecting Lp(a) concentrations. In our study, we minimized confounding factors by recruiting a group of presumably healthy individuals without acute or chronic diseases. Furthermore, a key strength of our study is the parallel analysis of Lp(a) concentrations in both adults and children from the same population, using the same Lp(a) assay method. Harmonization of Lp(a) results obtained with different immunoassays remains indeed a major challenge due to the potential impact of the heterogeneity of apo(a) isoforms and the variability in kringle IV number in Lp(a) quantification. In our study, Lp(a) measurements were performed using the Randox assay, that shows minimum apo(a) size related influence (*34*).

However, we recruited a relatively small number of study participants from a single region in north-central Poland; therefore, this group is not fully representative of the overall Polish population. It should be also noted that reporting Lp(a) results in nmol/L is considered more methodologically accurate, as it reflects the number of apo(a) molecules. However, assays that accurately measure Lp(a) in nmol/L are not commercially available. Commercial immunoturbidimetric and nephelometric assays use polyclonal antibodies that may recognize a variable number of kringle IV repeats, potentially leading to underestimation or overestimation of Lp(a) mass due to apo(a) size polymorphism (*1*, *35*). Among the available immunossays, the Randox Lp(a) assay (using Denka reagents) calibrated in mass units is considered to approximate molar measurements closely (*21*). Accordingly, Randox offers the possibility, for a limited number of analyzers, to convert instrument-specific Lp(a) calibrator values in nmol/L; however, this option is not available for the analyzer used in this study (Horiba ABX Pentra 400). Alternatively, conversion factors to convert Lp(a) in mass units to nmol/L that are available in the literature may be used. However, since an assay-specific conversion factor should be determined for each calibrator, the use of a single conversion factor may be not sufficiently accurate. For these reasons, we used mass units (g/L) to report Lp(a) results throughout the paper, and a single conversion factor 2.15 (widely accepted in the literature for the Randox method) was applied only for the calculation of non–Lp(a) apoB and for providing Lp(a) values in nmol/L (in parentheses) to allow comparison with mass units. These data should be however interpreted with consideration of potential inaccuracies (*19*, *36*, *37*).

In conclusion, this study reveals key age- and sex-related patterns in Lp(a) concentrations and their contribution to apoB and LDL-C concentrations. Approximately 20% of both adults and children had elevated Lp(a), with slightly lower concentrations observed in girls and a notable increase in women after age 40 and in men after age 60. Elevated LDL-C emerged as the strongest predictor of high Lp(a) concentrations in both women and children, underscoring its relevance in Lp(a)-related risk assessment. The %Lp(a)/apoB and %Lp(a)-C/LDL-C ratios increased in both sexes after age 50, accompanied by declining apoB and LDL-C concentrations and rising Lp(a) concentrations. This pattern suggests greater atherogenicity in older individuals, particularly in men. Notably, the highest relative Lp(a) contributions were observed in boys aged 9-11, despite low absolute lipid concentrations, indicating a potential early cardiovascular risk.

## Data Availability

The data will be shared on reasonable request to the corresponding author.
